# Adherence to a plant-based, high-fibre dietary pattern is related to regression of non-alcoholic fatty liver disease in an elderly population

**DOI:** 10.1007/s10654-020-00627-2

**Published:** 2020-04-22

**Authors:** Louise J. M. Alferink, Nicole S. Erler, Robert J. de Knegt, Harry L. A. Janssen, Herold J. Metselaar, Sarwa Darwish Murad, Jessica C. Kiefte-de Jong

**Affiliations:** 1 Department of Gastroenterology and Hepatology, Erasmus MC, University Medical Centre Rotterdam, Rotterdam, The Netherlands; 2 Department of Biostatistics, Erasmus MC, University Medical Centre Rotterdam, Rotterdam, The Netherlands; 3 Toronto Centre of Liver Disease, Toronto General Hospital, University Health Network, Toronto, Canada; 4 Department of Epidemiology, Erasmus MC, University Medical Centre Rotterdam, Rotterdam, The Netherlands; 5 Department of Public Health and Primary Care/LUMC Campus The Hague, Leiden University Medical Center, Postzone VO-P, Postbus 9600, 2300 RC Leiden, The Netherlands

**Keywords:** Hepatic steatosis, Dietary patterns, Mediterranean, Plant-based

## Abstract

Dietary lifestyle intervention is key in treating non-alcoholic fatty liver disease (NAFLD). We aimed to examine the longitudinal relation between well-established dietary patterns as well as population-specific dietary patterns and NAFLD. Participants from two subsequent visits of the Rotterdam Study were included. All underwent serial abdominal ultrasonography (median follow-up: 4.4 years) and filled in a food frequency questionnaire. Secondary causes of steatosis were excluded. Dietary data from 389 items were collapsed into 28 food groups and a posteriori dietary patterns were identified using factor analysis. Additionally, we scored three a priori dietary patterns (Mediterranean Diet Score, Dutch Dietary Guidelines and WHO-score). Logistic mixed regression models were used to examine the relation between dietary patterns and NAFLD. Analyses were adjusted for demographic, lifestyle and metabolic factors. We included 963 participants of whom 343 had NAFLD. Follow-up data was available in 737 participants. Incident NAFLD was 5% and regressed NAFLD was 30%. We identified five a posteriori dietary patterns (cumulative explained variation [R^2^] = 20%). The patterns were characterised as: *vegetable and fish*, *red meat and alcohol*, *traditional*, *salty snacks and sauces*, *high fat dairy & refined grains pattern*. Adherence to the *traditional* pattern (i.e. high intake of vegetable oils/stanols, margarines/butters, potatoes, whole grains and sweets/desserts) was associated with regression of NAFLD per SD increase in Z-score (0.40, 95% CI 0.15–1.00). Adherence to the three a priori patterns all showed regression of NAFLD, but only the WHO-score showed a distinct association (0.73, 95% CI 0.53–1.00). Hence, in this large elderly population, adherence to a plant-based, high-fibre and low-fat diet was related to regression of NAFLD.

**Electronic supplementary material:**

The online version of this article (10.1007/s10654-020-00627-2) contains supplementary material, which is available to authorized users.

## Introduction

Non-alcoholic fatty liver disease (NAFLD) is the most common liver disease worldwide and characterized by fat accumulation in the liver without the presence of the well-known risk factors for liver disease such as alcohol misuse and viral hepatitis [1]. The increase of NAFLD parallels the worldwide rise of non-communicable metabolic diseases, such as obesity and type 2 diabetes mellitus [2, 3]. In fact, it is predicted that NAFLD prevalence will continue to increase, which will subsequently lead to even more advanced liver disease and liver-related mortality in the coming years [4]. But apart from the liver-related sequelae, NAFLD is also regarded as the hepatic manifestation of the metabolic syndrome [5]. It is not only strongly associated with metabolic health, but it also actively contributes to the risk of cardiovascular disease incidence [6, 7].

Pathogenesis of NAFLD is multifactorial—there are many molecular pathways that contribute to the development of NAFLD—and it is likely that NAFLD pathogenesis differs between individuals [1]. A common denominator in the development of NAFLD, however, is diet. Furthermore, as there is no registered drug for the treatment of NAFLD, the mainstay of treatment is implementing a healthy diet and stimulating physical activity [8]. It has been repeatedly shown that weight loss of 5% or more of the total body weight is beneficial for liver health [9–11], meanwhile the Mediterranean diet has been shown to reduce liver fat independent of weight loss [12, 13]. To date, the Mediterranean dietary pattern is indeed regarded as the diet of choice for NAFLD [12]. And although there is a paramount of studies on the association between separate food items or groups with NAFLD, evidence on diet as a whole almost exclusively originates from either small or cross-sectional studies [12, 14–16]. Studying pre-hypothesized individual food items or nutrients can be of value, nonetheless, this approach has some important drawbacks [17]. For instance, one does not eat isolated nutrients but instead a complex mix of foods which can interact with each other, and moreover, foods are highly inter-correlated with each other. In addition, the effect of one nutrient can be small. Therefore, studying their separate effects is challenging in a real-life setting. Conceptually, the study of dietary quality—by means of dietary patterns—represents more information on dietary habits, food and nutrient consumption [18]. Recently, Ma et al. [19] showed for the first time, that high dietary quality, as assessed by the a priori hypothesized Mediterranean Diet Score (MDS) and the American Heart Eating Index, was associated with reduced odds of steatosis development in a large longitudinal cohort study.

Another way to study dietary patterns is the use of factor analysis to derive population-specific patterns, so-called a posteriori dietary patterns. To date, a few small cross-sectional studies appraised the association between a posteriori dietary patterns and steatosis. All identified an unhealthy dietary pattern that was associated with steatosis [16, 20, 21].

The combination of both a priori and a posteriori patterns might reveal a better understanding of the relation between dietary quality and NAFLD. The objective of this study was, therefore, to examine the relation between dietary patterns and NAFLD prospectively in an elderly predominantly Caucasian population. Specifically, we studied the following a priori dietary patterns: (1) the Mediterranean Diet Score, (2) the Dutch Dietary Guidelines, and (3) the World Health Organization recommendations. In addition, we identified five population-specific a posteriori patterns.

## Methods

### Study cohort

This study is embedded in the large ongoing population-based cohort study entitled the Rotterdam Study (RS). The RS commenced in 1989 and was designed to study elderly diseases as a response to the increasing proportion of elderly people in the population. A detailed description of the design and rationale has been described previously [22]. For the purpose of this study, we used two subsequent visits from the second cohort (RSII-3 and RSII-4). All individuals in this cohort were included based on their ZIP-code (being the suburb Ommoord in Rotterdam) and their age (at first inclusion 55 years or older).

We excluded participants that did not undergo ultrasound and participants with missing or unreliable food frequency questionnaires (FFQ), i.e. caloric consumption below 500 or above 7500 kilocalories (kcal) per day. We excluded unreliable transient elastography (TE), i.e. an interquartile range (IQR)/median liver stiffness measurement (LSM) > 0.30 in measurements with a median of ≥ 7.1 kilopascals (kPa) or above [23] and failure of TE (i.e. if no LSM was measured after at least ten attempts). Lastly, we excluded participants with well-known risk factors for steatosis, such as the presence of viral hepatitis (as measured by HbsAg and anti-HCV), the presence of alcohol misuse (measured using FFQs and defined as ≥ 2 units of alcohol per day in women and ≥ 3 units in men), or the use of pharmacy-registered drugs that are known to cause steatosis (i.e. tamoxifen, methotrexate, systemic corticosteroids, and amiodarone).

The RS has been approved by the institutional review board (Medical Ethics Committee) of the Erasmus MC University Medical Centre Rotterdam and by the review board of The Netherlands Ministry of Health, Welfare and Sports. Written informed consent was obtained from all participants.

### Food frequency questionnaires

Participants were asked to fill in a semi-quantitative 389-item FFQ, specifically developed for Dutch adults, during both research visits (RSII-3 and RSII-4) [24, 25]. This FFQ includes detailed questions on consumption over the last month and deals with type of food item, portion size, preparation method and frequency of consumption [26]. Questions within the FFQ are for example: “Did you eat eggs last month? If yes, how were they prepared (boiled or baked)? How often did you eat eggs per month (once or 2–3 times) or per week (once, 2–3, 4–5, 6–7 times)? How many eggs did you eat on an average day then?”. The 389 food items were grouped into 28 empirical food groups (supplementary Table 1), based on previous publications [20, 21, 27], and adapted based on the food item and group quantities (i.e. merged similar groups with a very low median intake).

### A priori dietary patterns

We chose to study the Mediterranean Diet Score (MDS), the Dutch Dietary Guidelines (DDG), and the World Health Organization (WHO) recommendations as a priori dietary patterns.

The MDS, first described by Trichopoulou et al. [28], originally has 9 components, of which 7 components are regarded beneficial (i.e. vegetables, legumes, fruits, nuts, whole grains, fish and mono-unsaturated fatty acids (MUFA)-to-saturated fatty acids ratio) and 2 components are regarded hazardous (i.e. red meat intake and excessive alcohol use). For the purpose of this study, we adapted the MDS by excluding the alcohol component from our calculation, as the MDS cut-off for hazardous alcohol use is 50 g per day, which is a very high cut-off in the context of a hepatic health outcome [29]. Moreover, alcohol consumption was included in the multivariable model as potential confounder. Hence, our adapted MDS has 8 components. All components were given a score of 0 (unhealthy) or 1 (healthy) based on sex specific median cut-offs, and summed up.

The DDG is a predefined index that was developed in 2015 and describes a general advice to follow a balanced and healthy dietary pattern [30]. The DDG is scored on the following points, consumption of (1) vegetables (≥ 200 g/day), (2) fruit (≥ 200 g/day), (3) whole-grain products (≥ 90 g/day), (4) legumes (≥ 135 g/week), (5) unsalted nuts (≥ 15 g/day), (6) fish (≥ 100 g/week), (7) dairy (≥ 350 g/day), (8) tea (≥ 150 mL/day), (9) whole grains ≥ 50% of total grains, (10) unsaturated fats and oils ≥ 50% of total fats, (11) red and processed meat < 300 g/week, (12) sugar-containing beverages (≤ 150 mL/day), (13) alcohol (≤ 10 g/day), and (14) salt (≤ 6 g/day).

We calculated the WHO-score based on the recent revised guidelines of the WHO (October 2018) [31]. This score is composed of 6 components which are scored as 0 if unhealthy and 1 if healthy. The components are scored as healthy if they satisfied the following criteria: (1) vegetables and fruit intake of ≥ 400 g per day, (2) sugar intake (added and sugar sweetened beverages) of < 10 g per day, (3) energy percentage from fat intake < 30%, (4) energy percentage from saturated fat intake < 10%, (5) energy percentage from trans fatty acid intake is < 1%, and (6) salt intake of < 5 g per day.

### A posteriori dietary patterns

A priori dietary patterns signify patterns described/identified in previous studies or specific habits of certain populations. They therefore do not necessarily ‘fit’ every population. For example, the Mediterranean Diet is natural for the Greek population in which this diet has been developed, whereas other populations such as the Dutch or Asian have different eating habits. We believe it is therefore of interest to also use a posteriori dietary patterns. These are population-specific dietary patterns and were derived using factor analysis on the 28 food groups at baseline with Varimax rotation and minimum residual estimation, using the function “fa” from the R package psych [32]. We included 5 dietary patterns based on the bend in the scree plot (supplementary Fig. 1). The factor loadings for each food group reflect the relationship between the food group and the respective factor (i.e. dietary pattern). Subsequently we calculated adherence scores, separately for both visits, by multiplying the factors (determined for the food groups at baseline) with the observed values of the food groups at baseline (RSII-3) and follow-up (RSII-4), respectively. Each score at baseline was scaled to have a mean zero and a standard deviation (SD) of one. The same scaling parameters were used for the corresponding score at follow-up, to optimize comparability.

### Hepatic imaging

For the purpose of this study, all participants underwent an abdominal ultrasound (Hitachi HI VISION 900) and TE (FibroScan®, EchoSens, Paris, France). Both examinations were performed at the same visit by an experienced nurse technician. The diagnosis of steatosis was dichotomized as yes or no, because of the poor sensitivity for the grading of steatosis but the good performance for diagnosing moderate/severe steatosis [33]. Steatosis was defined as hyperechogenic liver parenchyma as compared to the kidney parenchyma [34]. The practical performance of the transient elastography has been described in detail previously [35]. In short, both M and XL probe were available for the liver stiffness measurements (LSM) and used dependent on the subcutaneous fat layer as instructed by the manufacturer. Reliability criteria are described in the paragraph above (“Study Cohort”). Additionally, participants with an intra-cardiac device were excluded from the analyses. LSM were given as kilopascals (kPa). We used the previously proposed cut-off value of 8 kPa to proxy the presence of fibrosis in participants with steatosis, from this point forward referred to as non-alcoholic steatofibrosis (NASF) [36]. As the main focus of this present study is NAFLD, participants with an LSM of 8 kPa or higher without steatosis were excluded.

### Other covariates

All blood samples were drawn after overnight fasting. Automatic enzyme procedures (Roche Diagnostics GmbH, Mannheim, DE) were used to determine lipid profile, glucose, alanine aminotransferase, aspartate aminotransferase and gamma-glutamyltransferase. Insulin and viral hepatitis B or C were determined using an automatic immunoassay (Roche Diagnostics GmbH, Mannheim, DE). Detailed information on drug use was obtained via automated pharmacy linkage (with which 98% of the participants were registered). A 3 h home interview was carried out by trained research nurses and included questions on physical activity, smoking behaviour, education level, medical history, and demographics. In the research centre, anthropometrics were measured (i.e. weight, height, and waist and hip circumference), as well as blood pressure (median value after two measures in an upright position). The metabolic syndrome was defined using the harmonizing consensus criteria from Alberti et al. and contained 5 components on abdominal obesity, lipid profile, blood pressure, and fasting plasma glucose [37]. The comorbidities diabetes mellitus and hypertension were established on the basis of drug use for the respective comorbidity or findings at physical examination, as described in detail previously [38].

### Statistical analyses

Participant’s characteristics at baseline and follow-up are summarized using the median and first and third quartile, median and range (for dietary variables), or percentages.

To examine the association between the dietary patterns, micronutrient and macronutrient composition, we calculated and tested Spearman correlation coefficients between the raw values of (subtypes of) macronutrients as well as adjusted for total energy intake and the components of the a posteriori dietary patterns, and the adherence scores at baseline. Differences between energy-adjusted and energy & dietary pattern-adjusted correlation coefficients may be explained by overlapping characteristics of the different a posteriori patterns, which could outweigh each other’s effects.

In addition, as a supplementary analysis, we assessed the cross-sectional association between the different energy-adjusted food groups and NAFLD at baseline using univariable logistic regression. The energy-adjustment was carried out using the residual method [39].

To investigate the association of diet with NAFLD over time in the presence of missing values in the covariates we used Bayesian logistic mixed models, as implemented in the R package JointAI [40]. In this approach missing values in covariates are imputed simultaneously with the estimation of the regression coefficients of interest, and the added uncertainty in the coefficients due to the missing values is automatically taken into account [41, 42]. This imputation was done using the covariates of our most extensive set (i.e. Model 2, given below). Please find more information on the missing data and imputed values in the separate supplementary data file. The choice of a mixed model allowed us to include data from all patients that fulfilled the above mentioned inclusion criteria, even when no follow-up measurement was available. A random intercept was included in the mixed model to take into account correlation between repeated measurements within the same subject. Separate models were fitted for each of the a priori dietary patterns and the five a posteriori patterns. As the five a posteriori dietary patterns explain approximately 20% of the variation in dietary intake in the population, they were analysed together in one model.

Two sets of covariates were created. The first set (“Model 1”) contains baseline age (in years), physical activity (in metabolic equivalent task h/week) and education level (low/intermediate/high), and in addition, sex, energy intake (in kilocalories per day), alcohol consumption (in units per day), and follow-up time (in years). The second set (“Model 2”) additionally contains covariates that reflect potential confounding, colliding, or mediating factors, i.e. baseline type 2 diabetes mellitus, baseline hypertension, and BMI. To allow the effect of diet to change over time and to allow effect modification by BMI interaction terms between the respective dietary pattern variable(s) and follow-up time (in Model 1 and 2) and BMI (only in Model 2) were included.

To obtain results for Model 1, ten sets of imputed values were extracted from each of the analyses of Model 2, then Model 1 was fitted on each dataset. Output from the ten repeated analyses per model was combined to calculate overall results. Since none of the interaction terms mentioned above had relevant contribution to any of the models, and the presence of interaction terms in a model complicates the interpretation of the regression coefficients substantially, we re-fitted Model 1 and Model 2 without the interaction terms (using imputed values from the original models) and present only the results of these simplified models.

We also investigated the role of BMI as a mediating factor between diet and NAFLD: we performed additional analyses with BMI (continuous) as outcome measure. For this, Bayesian linear mixed models were used and incomplete covariates were again simultaneously imputed. The models contained the confounders from Model 2, an interaction term between the dietary patterns and follow-up time, and a random intercept.

We also examined adherence to dietary patterns in relation to NAFLD severity. Due to the low number of NASF patients, we were not able to perform mixed effects logistic regression models on this outcome. In order to gain insight into the association between dietary patterns and NASF, we therefore plotted the (a posteriori and a priori) dietary pattern adherence scores across participants with NASF, participants with ‘simple’ steatosis, and participants without steatosis.

We used non-informative priors for our Bayesian analyses. Results from the Bayesian analyses are presented as posterior means and 95% credible intervals (CI). All analyses were performed using R version 3.5.2 and the packages JointAI (version 0.5.1) and psych (version 1.8.12). More detailed information of the statistical analyses can be found in the supplementary methods.

## Results

### Participant characteristics

**Fig. 1:**
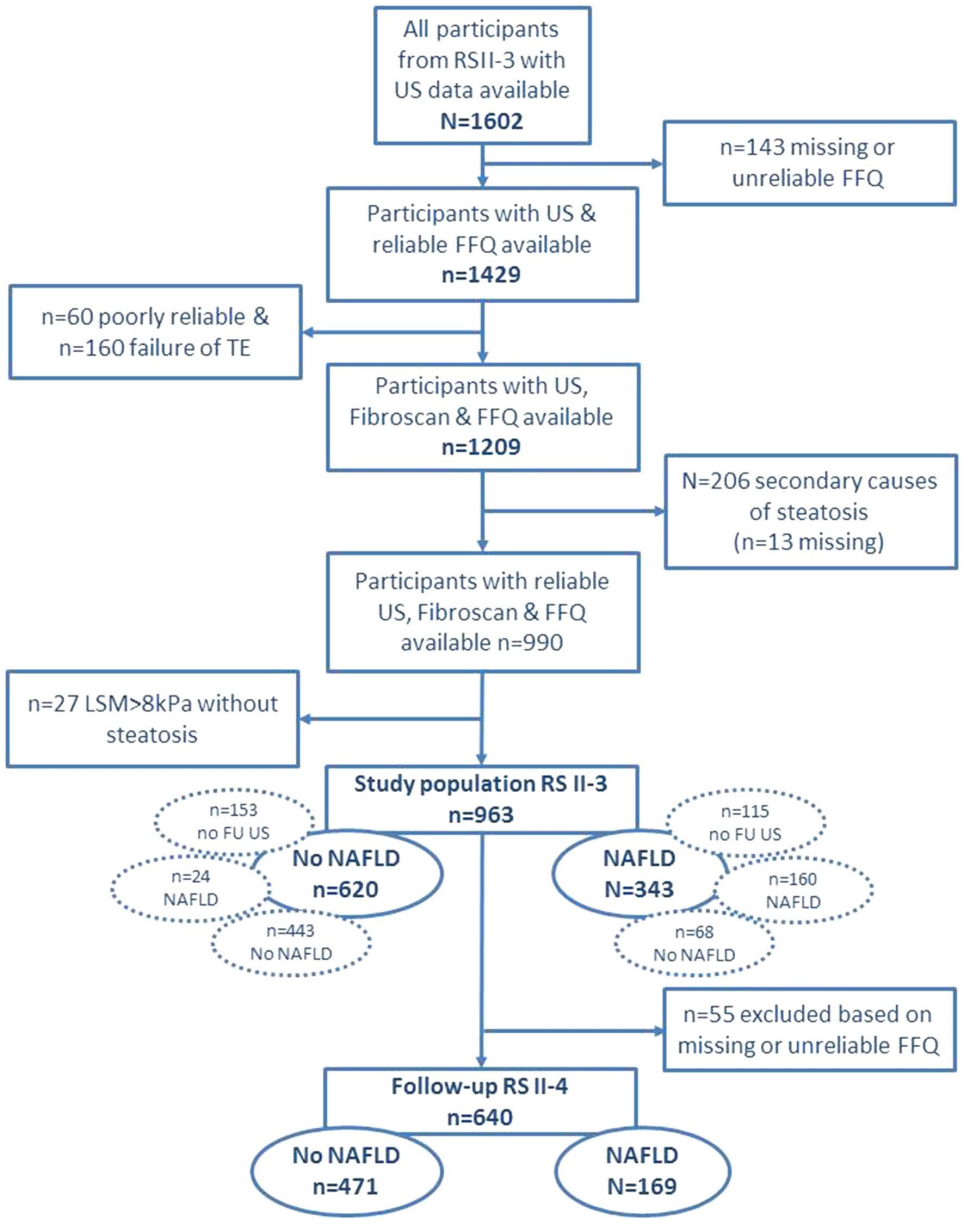
Flowchart of the study. The dotted encircled ovals depict the diagnosis at follow up RS II-4 for the ‘no NAFLD’ and ‘NAFLD’ group in RS II-3. The numbers of ‘No NAFLD’ and ‘NAFLD’ in RS II-4 depicts these encircled numbers minus the exclusion of 55 unreliable or missing FFQ at follow-up. Please find supplementary Table 2 for more information. *FFQ* Food Frequency Questionnaire, *FU* follow-up, *KPa* Kilopascals, *LSM* liver stiffness measurements, *NAFLD* non-alcoholic fatty liver disease, *RS* Rotterdam Study, *TE* transient elastography, *US* ultrasound

The flowchart of the study is illustrated in Fig. 1. After exclusion, 963 (60.1%) participants were eligible for this study at baseline. Prevalence of men (n = 424/963 vs n = 280/639) and steatosis (n = 343/963 vs n = 252/639) were similar in the included and excluded group (*P *= 0.93 and *P *= 0.12 respectively). BMI was slightly lower in the included group (mean 27.2 ± 3.8 kg/m^2^) than in the excluded (mean 28.0 ± 4.4 kg/m^2^) group (*P *< 0.01) as was the mean age (72.0 ± 4.8 in the included group and 72.7 ± 5.7 in the excluded group; *P *< 0.01). Of all included participants, 343 had NAFLD (35.6%), of which 31 (9%) had coincident elevated LSM, i.e. NASF (Table 1).

**Table 1:** Participant characteristics

	Baseline data n = 963	Follow-up data n = 640
Demographics		
Age (years)	71.0 [68.6, 73.4]	75.1 [72.7—77.4]
Female (%)	56.0	54.7
Caucasian (%)*	97.7	–
Education level (%)†		
Low/intermediate/high	48.2/31.7/20.1	–
Smoking status (%)‡		
Never/past or current	37.7/62.3	–
Alcohol (units/d)	0.49 [0.08, 1.22]	0.42 [0.04, 1.21]
Physical Activity (metEqh/wk)§	44.7 [18.0, 84.6]	–
Physical examination		
BMI (kg/m^2^)¶	26.9 [24.5, 29.4]	26.5 [24.3, 29.2]
Waist circumference (cm)	93.4 [84.8, 101.2]	94.3 [85.4, 101.5]
Biochemistry		
AST (U/L)	25.00 [21.00, 28.00]	–
ALT (U/L)	17.00 [14.00, 22.00]	–
GGT (U/L)¶	23.00 [17.00, 32.00]	–
Platelets (*10^9^/L)¶	254 [218, 301]	
HOMA-IR	2.68 [1.73, 4.14]	–
Total cholesterol (mmol/L)	5.40 [4.70, 6.20]	–
HDL-C (mmol/L)	1.44 [1.20, 1.72]	–
Triglycerides (mmol/L)	1.29 [0.98, 1.75]	–
Comorbidities		
Metabolic syndrome (%)	55.6	–
Diabetes mellitus (%)	15.2	–
Hypertension (%)	82.8	–
Liver imaging		
NAFLD (%)	35.6	26.4
Liver stiffness (kPa)	4.90 [4.00, 6.10]	5.10 [4.20, 6.10]
NASF (%)	3.2	1.6
Diet		
Energy intake (kcal/day)	1932 (1529, 2354)	1964 (1573, 2424)
MDS (range 0–10)	5.00 (4.00, 6.00)	5.00 (4.00, 6.00)
DDG (range 0–13)	7.00 (6.00, 8.00)	7.00 (6.00, 8.00)
WHO score (range 0–5)	2.00 (2.00, 4.00)	2.00 (2.00, 3.00)
Vegetable and fish pattern (SD)	− 0.19 (− 0.70, 0.56)	− 0.21 (− 0.80, 0.51)
Red meat and alcohol pattern (SD)	0.06 (− 0.53, 0.68)	0.03 (− 0.59, 0.57)
Traditional Pattern (SD)	− 0.05 (− 0.68, 0.60)	− 0.21 (− 0.86, 0.56)
Salty snacks and sauces pattern (SD)	− 0.13 (− 0.68, 0.57)	− 0.14 (− 0.67, 0.53)
High fat dairy and refined grains pattern (SD)	0.04 (− 0.54, 0.52)	0.04 (− 0.51, 0.54)

Data represents original non-imputed data as median [P25-P75], percentage, or median (range) for dietary data. Baseline data was complete except for *ethnicity data: missing in n = 179 cases, †education level: missing in n = 26 cases, ‡smoking status: missing in n = 55 cases, §physical Activity: missing in n = 68 cases, ¶covariables with < 0.5% missing values. Follow-up data was complete

*ALT* alanine aminotransferase, *AST* aspartate aminotransferase, *BMI* body mass index, *DDG* Dutch dietary guidelines, *GGT* gamma glutamyltransferase, *HDL-C* high density lipoprotein cholesterol, *HOMA-IR* homeostasis model assessment of insulin resistance, *MDS* Mediterranean diet score, *NAFLD* non-alcoholic fatty liver disease, *kcal* kilocalories, *metEqh* metabolic equivalent hours, *SD* standard deviation, *WHO* World Health Organization

Follow-up data of 737 participants (76.5%) was available, measured at a median time of 4.4 years [4.3—4.5] after the baseline measurement. Of those lost to follow-up, no participant was registered as deceased. Most participants had a similar ultrasound diagnosis at baseline and follow-up (Fig. 1). However, in the group without steatosis, 24 participants progressed to steatosis (5.1%), and in the group with steatosis, 68 participants regressed to no steatosis (29.8%). In supplementary Table 2 a detailed overview of follow-up by liver status is given.

Participant characteristics at baseline and follow-up are given in Table 1. In short, at baseline, median age was 71 [69–73] years, 56% were female, median BMI was 26.9 [24.5—29.4] kg/m^2^, and median LSM was 4.9 [4.0—6.1] kPa. At follow-up, median age was 75 [73–77] years, 55% was female, median BMI was 26.5 [24.3—29.2] kg/m^2^, and median LSM was 5.1 [4.2—6.1] kPa.

Adherence scores to all dietary patterns at baseline and follow-up are given in Table 1. In general, adherence to healthy eating patterns was similar and low. Median energy intake, as well as adherence to dietary guidelines was comparable at baseline and follow-up. In this population, absolute intake was quite low, the most consumed food groups were coffee (406 g or 1.6 cups/day), fruit (301 g/day), and vegetables (211 g/day) (Table 2). The energy-adjusted associations between food groups and NAFLD at baseline are given in supplementary Fig. 2.

**Table 2:** Median absolute intake and factor loadings for food groups per a posteriori dietary pattern

	Median [P25, P75] intake in grams	Food group factor loadings				
		Vegetable and fish pattern	Red meat and alcohol pattern	Traditional pattern	Salty snacks and sauces pattern	High fat dairy and refined grains pattern
Explained variation		4.8%	4.5%	4.3%	3.3%	2.5%
Fruit	301 [132, 529]	**0.292**	**− 0.225**	0.144		
Fruit juice	21 [0, 107]		− 0.105			**0.257**
Nuts	0.7 [0.0, 4.5]	0.110	− 0.116		**0.265**	
Vegetable oils and Stanols	27 [14, 43]		0.182	**0.543**		
Margarine or butter	11 [6, 19]		0.109	**0.391**		0.185
Tomatoes	18 [5, 35]	**0.586**	− 0.111		0.143	
Vegetables	211 [131, 325]	**0.584**		0.168		
Potatoes	68 [35, 102]		0.112	**0.314**		
Legumes	9 [0, 30]			0.142	**0.236**	
Whole grains	105 [68, 143]			**0.396**		− 0.196
Refined grains	30 [**14, 56**]	0.169	0.167			**0.346**
Egg products	13 [7, 21]	0.110	**0.229**			
Red meat	41 [23, 61]	0.196	**0.599**	**0.223**		0.174
Refined or organ meat	24 [13, 38]	0.177	**0.546**	**0.214**		0.167
Poultry	9 [4, 15]	**0.308**	0.185			
Fish	22 [10, 38]	**0.358**			0.141	
Low fat dairy products	193 [96, 319]	0.147		0.183		− 0.270
High fat dairy products	20 [7, 46]			0.114		**0.401**
Salty snacks	22 [10, 40]		**0.246**		**0.622**	
Sauces	2.7 [0.6, 6.3]	**0.267**	0.118		**0.480**	0.158
Sweet snacks or desserts	79 [48, 121]			**0.439**	0.181	**0.246**
Sugary drinks	0 [0, 43]		0.164		0.137	0.178
Diet soda or water	13 [0, 150]	0.191		− 0.149	0.123	
Tea	174 [54, 406]	0.138	− **0.308**	0.179		
Coffee	406 [174, 406]		**0.212**			
Wine	21 [0, 83]	0.186				
Beer or spirits	0 [0, 27]	− 0.105	**0.248**			
Soy products	0 [0, 0]		− 0.157		0.129	

Bold values reflect factor loadings above 0.2 or below – 0.2

### A posteriori dietary patterns

**Fig. 2:**
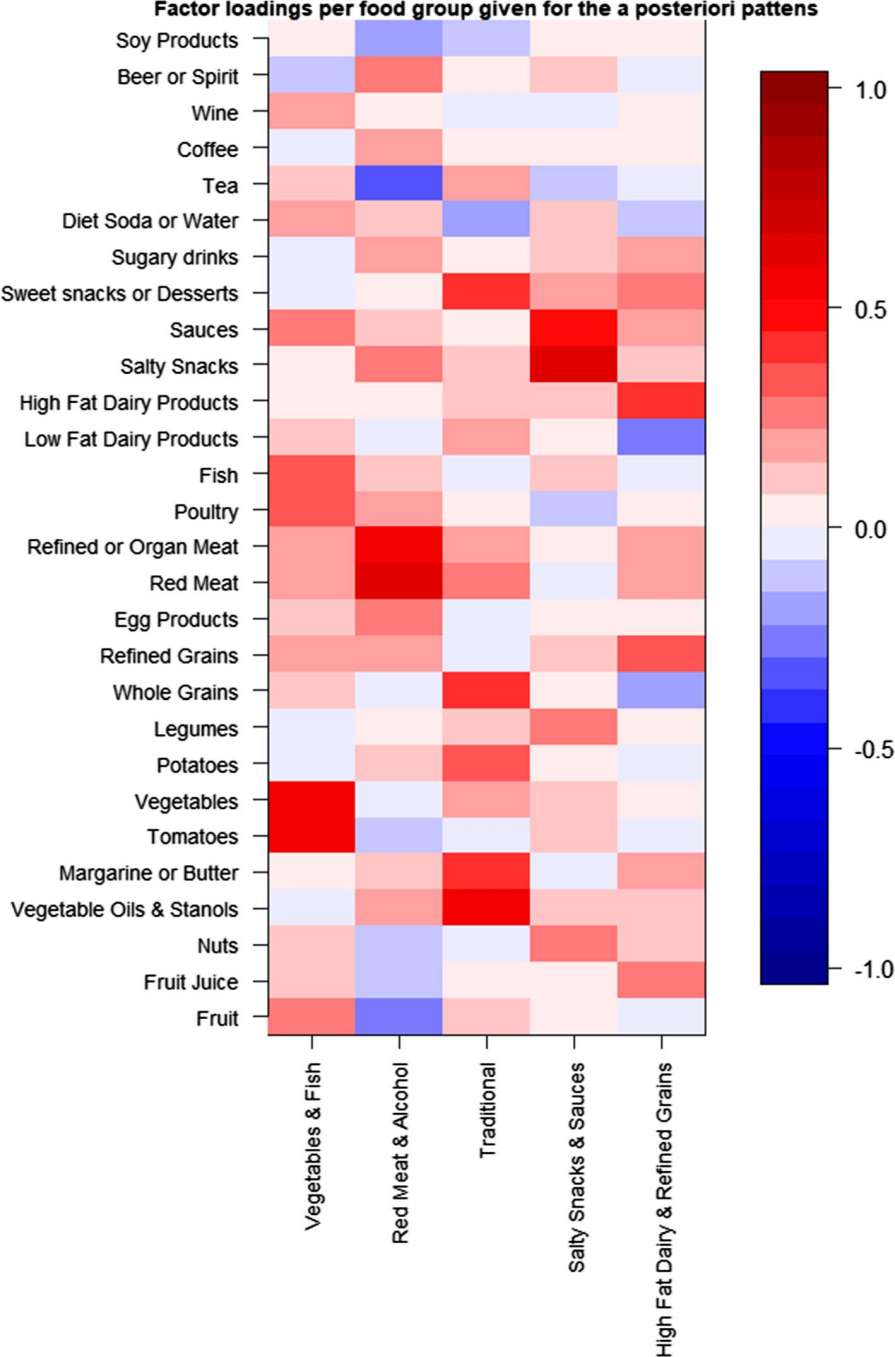
Heatmap of factor loadings from a posteriori dietary patterns. Factor loadings of a posteriori patterns per food group visualized using a heatmap. Red reflects a positive load, blue a negative load

The a posteriori patterns obtained by factor analysis are presented in Table 2 and Fig. 2. We identified five dietary patterns which together explained 19.5% of the variation in food intake. Specifically, the patterns explained respectively 4.8%, 4.5%, 4.3%, 3.3% and 2.5% of the variation in food intake. The first pattern was characterised by high intake of vegetables, poultry, fish, and fruit. This pattern was therefore named *vegetable and fish*. The second pattern was characterised by high intake of red, refined or organ meat, salty snacks, and beer or spirits, and low intake of fruit and tea. We therefore called this pattern *red meat and alcohol*. The third pattern was defined by high intake of vegetable oils and stanols and margarines or butters, potatoes, whole grains, and sweet snacks or desserts. This pattern was therefore called *traditional*. The fourth pattern was represented by high intake of savoury food groups such as nuts, legumes, salty snacks, and sauces. We thus named this pattern *salty snacks and sauces*. The last pattern was defined by high intake of fruit juice, refined grains, high-fat dairy products, and sweet snacks or desserts. This pattern was therefore called *high*-*fat dairy and refined grains.*

### Dietary pattern characteristics

**Fig. 3:**
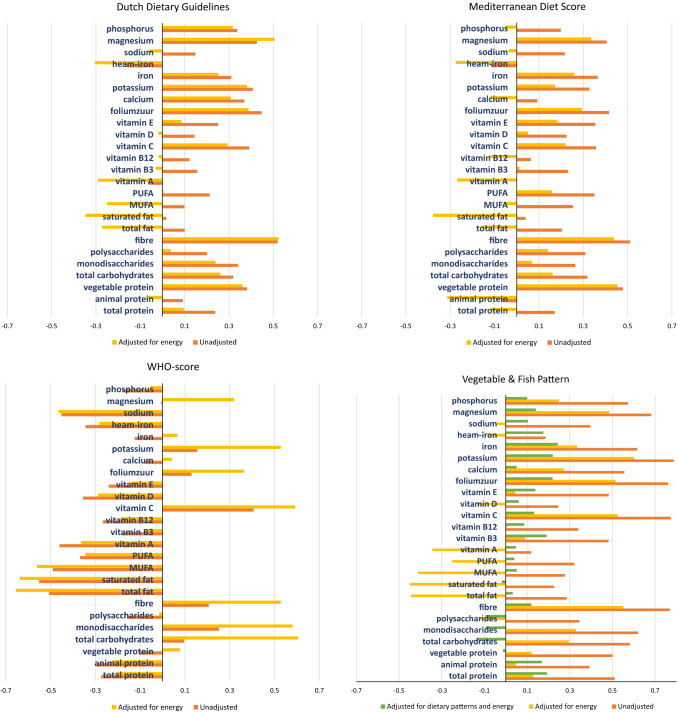

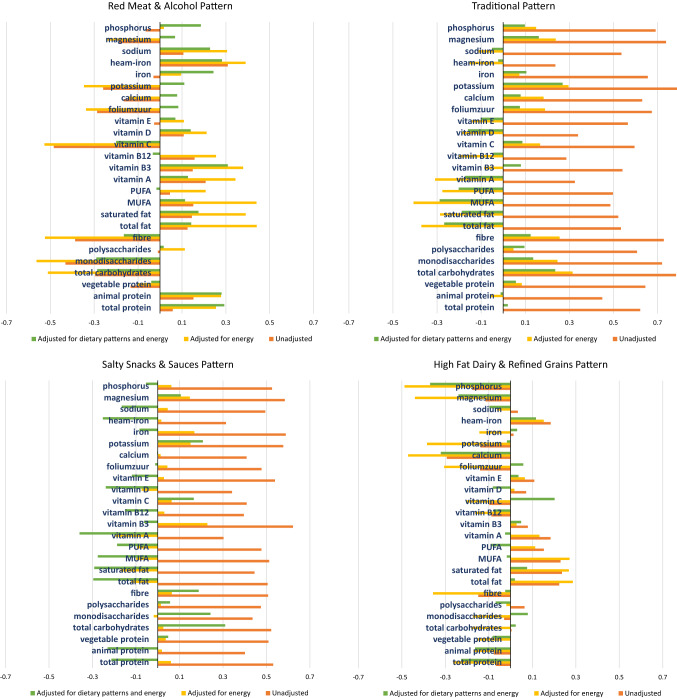
Correlation coefficients of adherence scores with standardized unadjusted, energy adjusted and energy and dietary pattern adjusted macronutrients and micronutrients. These figures reflect the Spearman correlation coefficients of various micronutrients and macronutrients per dietary pattern. The orange bars reflect the unadjusted correlation coefficients, the yellow bars the correlation adjusted for energy intake using the residual method, and the third green bar (in the a posteriori dietary patterns) reflects the correlation adjusted for other a posteriori dietary patterns and energy intake. *MUFA* mono-unsaturated fatty acid, *PUFA* poly-unsaturated fatty acid

In order to give more insight in the overall macronutrient and micronutrient composition of the dietary patterns, we performed Spearman rank correlation analyses at baseline (Fig. 3a–h).

The bars in orange reflect the unadjusted correlation coefficients and those in yellow the energy-adjusted coefficients. If the energy-adjusted correlation is lower than the unadjusted one, this indicates that, although the relative intake may be high, the absolute intake of the nutrient for that pattern is low. Which is, for example, the case in fat consumption for the DDG and the MDS (Fig. 3a, b). All a priori dietary patterns had a high energy-adjusted correlation with fibre and vegetable protein intake, and a negative correlation with overall fat intake (Fig. 3a–c). In addition, the DDG and the WHO-score had a relatively high correlation for mono-and disaccharide intake.

In Fig. 3d–h a third, green bar reflects the energy & dietary pattern-adjusted correlation. We added these results here since all patterns are analysed together in one regression model.

A posteriori patterns that resemble the healthy a priori dietary patterns most are the *traditional* and *salty snacks and sauces* patterns, which are also low in animal protein, fats, sodium and haem-iron, and high in vegetable protein, fibre, mono-and disaccharides and potassium (Fig. 3f, g). Whereas the *red meat and alcohol* and *high*-*fat dairy and refined grains* patterns were low in fibre and potassium and high in fats and haem iron (Fig. 3e, h).

### Relation between dietary patterns and NAFLD

A summary of the multivariable logistic mixed models between the a priori dietary patterns and risk of NAFLD are given in Table 3. Results indicate regression of NAFLD in all patterns, but only with an unambiguous credible interval for the WHO-score after full adjustment for sociodemographic, lifestyle and metabolic confounders (Table 3; OR 0.73, 95% CI 0.35–1.00).

**Table 3:** Summary of parameters of interest from logistic mixed models for risk of NAFLD with adherence to a priori dietary patterns

	Risk of NAFLD	
	Model 1	Model 2
	OR (95% CI)	OR (95% CI)
A priori dietary patterns		
MDS	0.86 [0.70, 1.07]	0.84 [0.66, 1.05]
DDG	0.88 [0.71, 1.08]	0.89 [0.71, 1.12]
WHO-score	0.74 [0.55, 0.99]	0.73 [0.53, 1.00]

This table reflects six different mixed logistic regression models. Please find supplementary Table 3 for results on all coefficients from these models. Numbers in bold reflect a tail-probability of < 0.05

Model 1 is adjusted for: sex, age, baseline education level, baseline physical activity, energy intake, alcohol intake, and follow-up time

Model 2 is additionally adjusted for BMI, baseline type 2 diabetes mellitus and baseline hypertension

*MDS* mediterranean diet score, *CI* credible interval, *DDG* Dutch Dietary Guidelines, *NAFLD* non-alcoholic fatty liver disease, *WHO* World Health Organization

The multivariable associations between a posteriori dietary patterns and risk of NAFLD is shown in Table 4. Adherence to the *traditional* dietary pattern was associated with regression of NAFLD after full adjustment (Table 4; OR 0.40, 95% CI 0.15–1.00) and adherence to the *salty snacks and sauces* pattern showed a similar result (OR 0.43, 95% CI 0.17–1.04).

**Table 4:** Logistic mixed model for risk of NAFLD with adherence to a posteriori dietary patterns

	Risk of NAFLD	
	Model 1	Model 2
	Mean (95% CI)	Mean (95% CI)
Follow-up time (years)	**0.74 [0.66, 0.84]**	**0.70 [0.60, 0.81]**
Age (years)*	0.93 [0.82, 1.04]	**0.88 [0.77, 1.00]**
Sex (female)	**0.17 [0.04, 0.57]**	0.21 [0.05, 0.78]
Energy intake (kcals)	**1.00 [1.00, 1.00]**	**1.00 [1.00, 1.00]**
Education level*		
Low	Ref	Ref
Intermediate	**0.24 [0.06, 0.84]**	0.35 [0.09, 1.33]
High	**0.06 [0.01, 0.30]**	**0.12 [0.02, 0.58]**
Physical activity* (metEqhs)	**0.99 [0.97, 1.00]**	1.00 [0.99, 1.01]
Alcohol intake (units)	0.85 [0.52, 1.40]	1.06 [0.61, 1.85]
BMI (kg/m^2^)	**–**	**3.01 [2.28, 4.19]**
Diabetes mellitus*	**–**	**41.5 [8.49, 273]**
Hypertension*	**–**	**7.93 [1.60, 50.8]**
*A posteriori dietary patterns*		
Vegetable and fish pattern	1.88 [0.90, 4.05]	1.31 [0.57, 3.01]
Red meat and alcohol pattern	1.40 [0.70, 2.80]	0.79 [0.36, 1.69]
Traditional pattern	**0.33 [0.14, 0.77]**	**0.40 [0.15, 1.00]**
Salty snacks and sauces pattern	0.63 [0.28, 1.35]	0.43 [0.17, 1.04]
High fat dairy and refined grains pattern	0.82 [0.55, 1.22]	1.23 [0.79, 1.96]

This table shows results (OR and 95% CIs) of two multivariable logistic mixed models

*BMI* body mass index, *CI* credible interval, *NAFLD* non-alcoholic fatty liver disease, *kcal* kilocalories, *metEqh* metabolic equivalent hours

*Depicts baseline variables. Numbers in bold reflect a tail-probability of < 0.05

Factors that were independently associated incidence of NAFLD were incremental energy intake, BMI, baseline diabetes and baseline hypertension. Factors that were associated with regression of NAFLD were female gender, follow-up time, and high education level (Table 4 and supplementary Table 3a–c).

### Additional analyses for BMI

In supplementary Table 4 we show the association between adherence to a posteriori and a priori dietary patterns with BMI over time. Taking interactions into account, BMI over time depended on the adherence of DDG and WHO-scores (i.e. low scores: BMI decreased, high scores: BMI increased). For MDS the interaction effect was so small that irrespective of the dietary score BMI generally increased over time. At baseline, none of the dietary patterns was associated with BMI, except for a priori pattern WHO which was associated with a lower BMI (supplementary Table 4d). In the model using a posteriori dietary patterns the effect of time on BMI depended on the score of the *high fat dairy & refined grains pattern* pattern. For low scores in this pattern the model estimated that BMI increased over time, whereas for high scores a slight decrease of BMI was found (supplementary Table 4a). On the other hand, there was a significant positive interaction between follow-up time and DDG and between follow-up time and WHO, which means the that BMI increased over time when adhering to these patterns and decreases for participants that had low scores on these patterns (supplementary Table 4c and d).

### Visualization of dietary patterns for NAFLD severity

Adherence to the different dietary patterns are visualized for NAFLD severity in supplementary Fig. 3. There was no particular difference in adherence between participants without steatosis, participants with simple steatosis and participants with NASF visible.

## Discussion

The first step in treating NAFLD is lifestyle modification and an important pillar in this treatment is diet. Although there is a paramount of studies on nutrition and NAFLD, there is no study yet that assesses the longitudinal association between well-known dietary quality scores, population-specific dietary pattern scores and NAFLD adjusted for important covariates such as BMI and metabolic confounders. We, therefore, examined both a priori and a posteriori dietary patterns in relation to NAFLD using comprehensive multivariable logistic mixed regression models in a large elderly white population-based cohort. After a follow-up period of almost 4.5 years, only 5% of the participants had incident NAFLD, whereas as much as 30% had regression of NAFLD. Amongst the three pre-hypothesized (a priori) dietary quality scores and the five a posteriori population-specific dietary patterns, two dietary pattern scores were associated with a lower risk of NAFLD, i.e. the WHO-score and the *Traditional* dietary pattern score. The latter was characterized by high intake vegetable oils & stanols, margarines or butters, potatoes, whole grains, and sweet snacks or desserts.

The three a priori dietary patterns that we studied in this paper –the Mediterranean diet, the Dutch dietary guidelines, and the world health organization diet score– largely overlapped in macro and micronutrient composition. All were highly correlated with fibre, vegetable protein, carbohydrate intake and potassium, and all were inversely correlated with animal protein, fat, sodium and haem iron intake. The only exception was the MDS, which was highly correlated with fat by its characteristic unsaturated fatty acid intake. Of the three patterns MDS is the one best-studied in relation to liver health, and accumulating evidence indeed suggests that this diet is beneficial for metabolic and liver health [12, 43]. Even though the evidence for MDS as therapeutic strategy for NAFLD predominantly originates form observational studies, the EASL consensus guidelines for NAFLD advocates the implementation of this diet as therapeutic strategy [8]. Our results are consistent with a beneficial effect of adherence to the MDS. To date, only one other longitudinal study in dietary quality has been performed [19]. This study from Ma et al. showed that adherence to the MDS was significantly associated with reduced risk of steatosis within the Framingham Heart Study. Important strengths of this study are the large sample size and the use of computed tomography. A limitation, on the other hand, was the fact that regression models were not adjusted for metabolic covariates such as diabetes and hypertension—of which we know and show in this paper—are strongly related to both NAFLD and diet.

To our knowledge, only few studies have examined the association between diet and NAFLD using factor analyses [16, 20, 21]. One of them was the cross-sectional study of Koch et al. that used 38 food groups and found an a posteriori pattern that was high in soups, beer, wine, juice, poultry and eggs and was associated with steatosis as assessed by MRI [20]. Another cross-sectional study identified four different a posteriori patterns (i.e. a *fast*-*food*, *prudent*, *high protein*, and *unsaturated fatty acid pattern*) using 15 food groups, and found that only the *fast*-*food* pattern was associated with ultrasound-diagnosed NAFLD [21]. Lastly, there has been one longitudinal study in adolescents (n = 995) which identified two a posteriori patterns, a *healthy* and a *western/unhealthy* pattern. This study found that adherence to the *western* dietary pattern at 14 years of age was associated with higher odds of NAFLD at 17 years. We did not find a detrimental association between dietary patterns and NAFLD, again, possibly because of the low number of incident NAFLD cases over time. But another important difference between our study and the others is that we adjusted for BMI, whereas they had either not taken BMI into account [20, 21], or found that their results dissipated after correction for BMI [16]. The only a posteriori pattern in our study for which we found a clear association was the *traditional* pattern. Interestingly, this diet resembled the macronutrient & micronutrient composition of the three a priori healthy diet scores, i.e. having a high correlation with fibre intake, mono-and disaccharide, vegetable protein, and potassium intake and an inverse correlation with fat, haem iron, sodium, and animal protein intake.

The rationale to use a posteriori dietary patterns is that they reflect the actual population-based dietary eating habits and may therefore reveal new insights in the relation between diet and disease in a particular population [17]. Together the five a posteriori dietary patterns explained almost 20% of the variation in food intake, but their independent explanatory ability was less than five percent. In addition, the five identified patterns were quite heterogeneous, which complicates unambiguous interpretation. Indeed appearing paradoxical results were obtained when comparing factor loadings and macro- and micronutrients correlation coefficients. For example, the factor loadings for the food group red meat is above 0.2 in the *traditional* pattern, whereas the macronutrient animal protein is negatively correlated to this pattern. There are two possible explanations for this paradox; (1) absolute intake of the food group red meat is low (median 41 g /day), which is supported by the difference in unadjusted and energy-adjusted correlation coefficients; and (2) the food group red meat is even more prominent in the *red meat and alcohol* pattern, as both patterns were adjusted for one another in the mixed logistic regression models, red meat is therefore not a unique feature of the *traditional* pattern. In other words, another combination of food groups may be more distinctive for the traditional pattern than red meat. The first explanation also reflects why adjusting for energy intake is important in this elderly population in which absolute intake is generally low. Indeed, semi-quantitative FFQs cannot measure exact energy intake and it is therefore important to look at the relative food intake. However, a diet high in energy-dense foods may not reflect the effect of the actual foods themselves, but rather an association with the high energy intake [39]. That is why we chose to show both energy-adjusted and unadjusted results.

An important characteristic of the healthy dietary patterns was fibre. Recently, a systematic umbrella review and meta-analyses, showed that high intakes of dietary fibre—from 25 g per day onwards—could prevent non-communicable diseases in the general population [44]. In addition, another population-based study showed that adherence to a plant-based diet promoted an increase in fibre-degrading bacteria in the gut. In that study, high adherence to the MDS (defined as the highest population-specific tertile in the study) was related to higher levels of short-chain fatty acid production in the faeces [45]. Indeed, butyrate production, an important short-chain fatty acid, is considered to be anti-obesogenic and has been proposed to decrease gut permeability and affect insulin sensitivity [46]. Additionally, in a recent study of our group we show that (in a larger subset of the population) animal protein was the only macronutrient that was associated with higher prevalence of NAFLD [38]. This finding is in line with other recent studies that demonstrate a detrimental effect of (particularly red) meat on liver health, insulin sensitivity, and overall mortality [47, 48]. In a spin-off study we examined whether diet-dependent acid load could explain the association between an animal protein-rich diet and NAFLD [49]. In this study, we found that an acidic diet was associated with high animal protein intake and low fruit intake as well as with NAFLD. An alkaline diet is generally accompanied by high potassium intake, which is an alkalinizing micronutrient [50]. The healthy dietary patterns in this study all had a positive correlation with potassium.

Although a major strength of our current study is that diet is taken into account as a whole, we cannot exclude the possibility that the associations found between dietary patterns and NAFLD are driven by particular food groups instead of by the entire dietary pattern [17]. The pattern *salty snacks & sauces*, for example, are characterised by “unhealthy” food groups that have a high sodium load, such as salty snacks and sauces. However, this pattern is also characterised by food groups that can exert specific beneficial effects on metabolic health, such as nuts and legumes [51]. Indeed, we have previously shown that specific food items, such as coffee and tea, are associated with NAFLD independent of dietary quality [52]. On the other hand, specific constituents within the diet can also be detrimental, such as haem iron from meat, which is associated to oxidative stress and insulin resistance [53]. Noteworthy is that both beneficial dietary patterns, the WHO-score and the *traditional* dietary pattern, had a negative correlation with vitamin E, an anti-oxidant that has been proposed as treatment of NAFLD [8]. But whereas vitamin E has been proposed to exert anti-oxidant beneficial effect on the liver [54], vitamin E has also repeatedly been associated with detrimental health outcomes such as haemorrhagic stroke [55] and mortality [56].

The large sample size and prospective design of our study enabled us to include important sociodemographic, lifestyle and metabolic confounders in the analysis. Nonetheless, there are several limitations that need mentioning. This cohort comprises an elderly, almost exclusively Caucasian population, which might hamper generalization towards other populations. Also, although none of the participants was deceased at time of follow-up, we cannot exclude the possibility that follow-up measurements were missing *not at random* during follow-up. In addition, although we have a large study cohort of almost a thousand participants, only a small number of incident NAFLD and regressed NAFLD was found. This might have reduced our power to detect relevant effects. In line with this, the number of NASF cases was also quite small which hindered us from performing multivariable analyses on this phenotype. However, the number of NASF cases that we found in our study is in line with the prevalence of elevated LSM in a general NAFLD population [57]. Also, the presence of steatosis could have influenced LSM, we therefore used a conservative cut-off proposed for patients with severe steatosis. This could have led to underestimation of individuals with significant fibrosis. But even with this conservative cut-off no evident difference in adherence to dietary patterns was found [36]. In addition, ultrasound and transient elastography as diagnostic tool for steatosis is not the gold standard, ultrasound is a subjective measure with a sensitivity of 79.7% and specificity of 86.2%. Diagnosis of steatosis could be subject to time of measure and operator [58]. Liver biopsy is the gold standard. However, it is ethically debatable and practically infeasible to perform an invasive procedure such as the liver biopsy in large cohorts. On the other hand, all our ultrasound measurements were performed by a single operator, which reduces the risk of bias. Furthermore, transient elastography over time is not validated in patients with NAFLD as of yet, but in hepatitis B longitudinal assessment of fibrosis using transient elastography is being used [59]. Moreover, the use of food frequency questionnaires could have led to recall bias as the questions concern dietary behaviour over the last month. In particular non-differential measurement error could have occurred (i.e. over or underreporting, as reflected in total energy intake). We have, however, dealt with the potential of this type of bias by adjusting for energy intake using the residual method and by adjusting for energy intake in the multivariable analysis to account for the extraneous variation in energy intake. In addition, previous studies have confirmed that FFQs provide sufficient information on diet to study overall dietary quality [60]. Also, BMI in elderly is a suboptimal measure for adiposity, we therefore cannot exclude the possibility that a decrease in BMI is caused by a loss in muscle mass rather than fat mass, which is generally an expression of poor nutritional and physical health [61]. Indeed, if adherence to a presumed healthy diet is low, this could also be explained by low overall food intake rather than imply that overall dietary quality is poor. In this case, low adherence to healthy diets could lead to weight loss. As the absolute consumption is low, snacking might not be detrimental at all in this frail elderly group. In line with this, as our results comprise an elderly white population, a word of caution on generalisability is therefore warranted.

In summary, in this large prospective elderly epidemiological study we found that adherence to the World Health Organization Diet and to a *Traditional* diet—both characterized as plant-based, high-fibre and low-fat- was related to regression of NAFLD over time. This finding is in line with increasing evidence that (red) meat is negatively associated with NAFLD and other metabolic comorbidities [47, 48]. However, we are the first longitudinal study to examine full dietary patterns with adjustment for BMI and energy intake in relation to NAFLD. While adding important new information, we believe that at the current moment, without external validation of our results and with the unknown generalizability outside our elderly population, it is too premature to firmly adapt guidelines or clinical management accordingly. Future (randomized) interventional studies on for example a meat-based vs no meat diet, ideally in younger populations, are needed. Also, there is still a gap in knowledge on the potential underlying mechanisms, such as dietary acid load or metagenomic alterations in the blood that effectuate the steatogenic effect of diet on the liver. On the other hand, to the best of our knowledge a diet that is rich in fibre and vegetables and low in red meat hasn’t been described to be detrimental for health either. And with the alarming rise in NAFLD incidence in children [62], clear and effective recommendations on the treatment of NAFLD are desperately needed. We therefore think that adherence to such a diet while awaiting the results of future studies may be beneficial.

## Electronic supplementary material

Below is the link to the electronic supplementary material.

Supplementary material 1 (DOCX 233 kb)

Supplementary material 2 (PDF 273 kb)

## Abbreviations

BMI: Body mass index
CI: Credible interval
DDG: Dutch dietary guideline
FFQ: Food frequency questionnaire
KPa: Kilopascals
LSM: Liver stiffness measurements
MDS: Mediterranean diet score
MUFA: Mono unsaturated fatty acids
NAFLD: Non-alcoholic fatty liver disease
NASF: Non-alcoholic steatofibrosis
RS: Rotterdam study
SD: Standard deviation
TE: Transient elastography
WHO: World Health Organization
