# Supplementary material for: Adherence to a plant-based, high-fibre dietary pattern is related to regression of non-alcoholic fatty liver disease in an elderly population
Source: Eur J Epidemiol. 2020 Apr 22;35(11):1069–85. doi: 10.1007/s10654-020-00627-2 (PMC7695656; doi:10.1007/s10654-020-00627-2)
Supplement: Supplementary file 1 — Supplementary material 1 (DOCX 233 kb) [file 10654_2020_627_MOESM1_ESM.docx]

# Supplementary Methods:

For our main analyses we used the R package JointAI (40) which performs simultaneous analysis and imputation using the Bayesian framework. We opted for the Bayesian method as opposed to "standard" mixed models since the latter suffered from numerical problems that prevented us from obtaining any results. Moreover, the Bayesian approach has been shown to be superior for handling incomplete covariates in the presence of longitudinal data and non-linear associations (such as interaction terms), compared to standard implementations of multiple imputation (the current gold-standard for handling missing covariate values).(42, 63, 64)

In the method used here, in addition to the analysis model of interest (the logistic mixed model for NAFLD), models are specified for each of the longitudinal covariates (energy intake, alcohol intake, BMI (1 value missing at follow-up) and the dietary pattern variables) and each of the incomplete baseline covariates (socioeconomic status (26 missing values) and physical activity (68 missing values). These additional models allow us to obtain imputed values for missing covariate values. The choice of models used for the covariates depends on the type of each of the variables. Specifically, we used

- a cumulative logit model for socioeconomic status (ordered factor with three categories),
- a gamma regression model with a log-link for physical activity (right-skewed continuous),
- a gamma mixed regression model with a log-link for alcohol intake (right-skewed continuous, measured at baseline and follow-up), and
- linear mixed models for energy intake, BMI and each of the dietary pattern variables.

The analysis model of interest and covariate models are estimated jointly so that the imputed values of incomplete covariates are used directly in the main analysis model.

To use all available information for the imputation, and since the different dietary pattern variables contain overlapping but not identical information, we included all dietary pattern variables in the models for covariates. For example, during the analysis of the association of NAFLD with the DDG pattern, the other dietary pattern variables (WHO, MDS and the five a-posteriori patterns) were not included in the logistic mixed model for NAFLD, but all eight variables were included in the models for the covariates.

For the same reason (to utilize all information available) the most extensive model ("Model 2"), including both interactions (dietary pattern with time, and dietary pattern with BMI) was used to extract imputed values which were then used in the estimation of the smaller models (models without interactions and/or models with the smaller set of confounders).

In the Bayesian framework, the estimation of regression coefficients is typically performed using the Markov Chain Monte Carlo (MCMC) method. In simple terms, this is an iterative procedure in which a chain of values is created for each regression coefficient and each missing value. The values in such a chain represent samples of the distribution of the respective coefficient or missing value. Results of a Bayesian regression model are obtained by summarizing this sample using the mean and 2.5% and 97.5% quantiles, which form the credible interval.

To obtain imputed values, random draws from the chains for missing values were performed. The values from each draw were then filled into the original incomplete data, thereby creating multiple completed versions of the data.

Each completed dataset was analysed to obtain results for "Model 1" as well as the models without interaction terms. To obtain overall results from these models, pooled over the multiple versions of the completed data, the chains resulting from each of the analyses were merged and the summary (mean and quantiles) calculated from the merged sample.


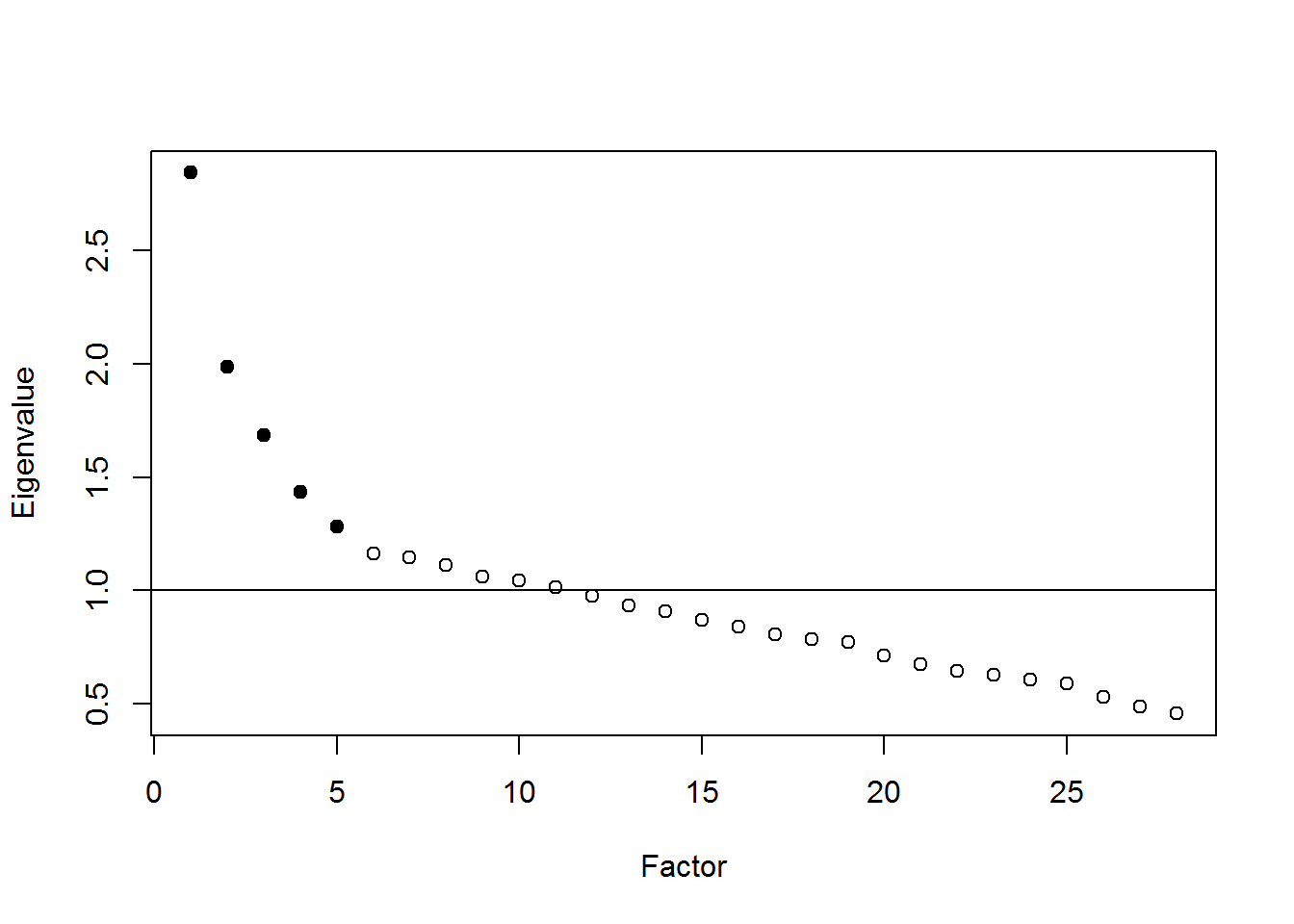


# Supplementary Figure 1: Scree plot of the factor analysis

The y-axis reflects the Eigenvalue and the x-axis the derived factors. The filled dots reflect the factors that we included in this manuscript as a-posteriori patterns

# Supplementary Table 1: food items in food groups

| **Food group** | **Included food items** |
| --- | --- |
| Fruit | fruit miscellaneous, apples, bananas, pears, oranges, strawberries, grapes |
| Fruit juice | orange juice, other fruit juices |
| Nuts | (mixed) nuts, flax seed |
| Vegetable Oils & Stanols | diet/low-fat/fluid margarine or frying-fat, gravy, low-fat butter, full-fat/low-fat stanoil margarine, vinegar-oil, olive oil, mono or polyunsaturated fatty acid rich oil |
| Margarines or Butters | full-fat butter/margarine/frying-fat, lard, gravy n.o.s., spreads n.o.s. |
| Tomatoes | tomato sauce, ketchup, tomatoes |
| Vegetables | spinach, cauliflower, broccoli, cabbage, carrots, vegetable soup, wok vegetables, onions, beans, lettuce, cooked vegetables, vegetable juice, raw vegetables, salads |
| Potatoes | potatoes (without fat), oven fries, mash potato |
| Legumes | legumes, legume soup |
| Whole Grain Products | muesli, fibre-rich breakfast cereals/ knackerbrod, rye bread, brown rice/rolls/bread, whole-grain bread/rolls, whole-grain dough, whole grain rusk, multigrain rolls/bread |
| Refined Grain Products | biscuits, cornflakes, white rolls/bread/dough/rice, toasts |
| Eggs | eggs (baker or boiled) |
| Red Meat | ham, minced meat, pork chop, beef, meatball, satay |
| Refined or Organ Meat | bacon, gammon, meat n.o.s., rolled fillet, miscellaneous meat, smoked sausage, cold cuts, cooked liver, liver products, organ meat n.o.s., liver sausage |
| Poultry | chicken |
| Fish | mussels, European flounder, salmon, trout, herring, fish n.o.s. |
| Low-fat Dairy | fat-free/low-fat yoghurt or quark, low-fat cheese (20-30%), fat-free/low-fat (pasteurized) milk, buttermilk |
| Full-fat Dairy | full-fat yoghurt, full-fat cheese (40+), cheese n.o.s., whipped cream, cheese fondue, cream, full-fat (pasteurized) milk, coffee cream, creamer |
| Salty Snacks | peanutbutter, peanuts, peanut sauce, pizza, fries, snackbar products, spring rolls, crisps, salty salad snack |
| Sauce | halvanaise, mayonaise, warm sauces, sauce/dressing n.o.s. |
| Sweet extras or Desserts | chocolates (dark/milk/white), sugar (additives), candybar, sweets, mousse, custard, pie, icecream, rusk, croissants, currant bun, *ontbijtkoek*, pancake, sweet biscuits, cake |
| Sugary Drinks | Ice pop, soda, breakfast drinks, fat-free/full-fat/low-fat chocolate milk |
| Diet Soda or Water | diet soda, mineral water |
| Tea | herbal, black and green tea |
| Coffee | coffee |
| Wine | red wine, white wine |
| Beer or Spirit | beer, low-alcohol beer, strong liquors, *advocaat* |
| Soy Products | bean curd, meat substitutes, soy dessert, soy milk |

Food items in *italic* reflect typical Dutch food items of which there is no sufficient translation

**Abbreviations:** n.o.s.: not otherwise specified

# Supplementary Table 2: Follow-up data according to liver status

|  | no NAFLD* | NAFLD without elevated LSM^†^ | NAFLD with elevated LSM^‡^ | lost to follow-up |
| --- | --- | --- | --- | --- |
| no NAFLD | 443 | 23 | 1 | 153 |
| NAFLD without elevated LSM | 62 | 144 | 4 | 102 |
| NAFLD with elevated LSM | 6 | 6 | 6 | 13 |

*40 additional participants without reliable FFQ available. †14 additional participants without reliable FFQ available. ‡1 additional participants without reliable FFQ available.

**Abbreviations:** FFQ: food-frequency questionnaire**,** LSM: liver stiffness measurement, NAFLD: non-alcoholic fatty liver disease.


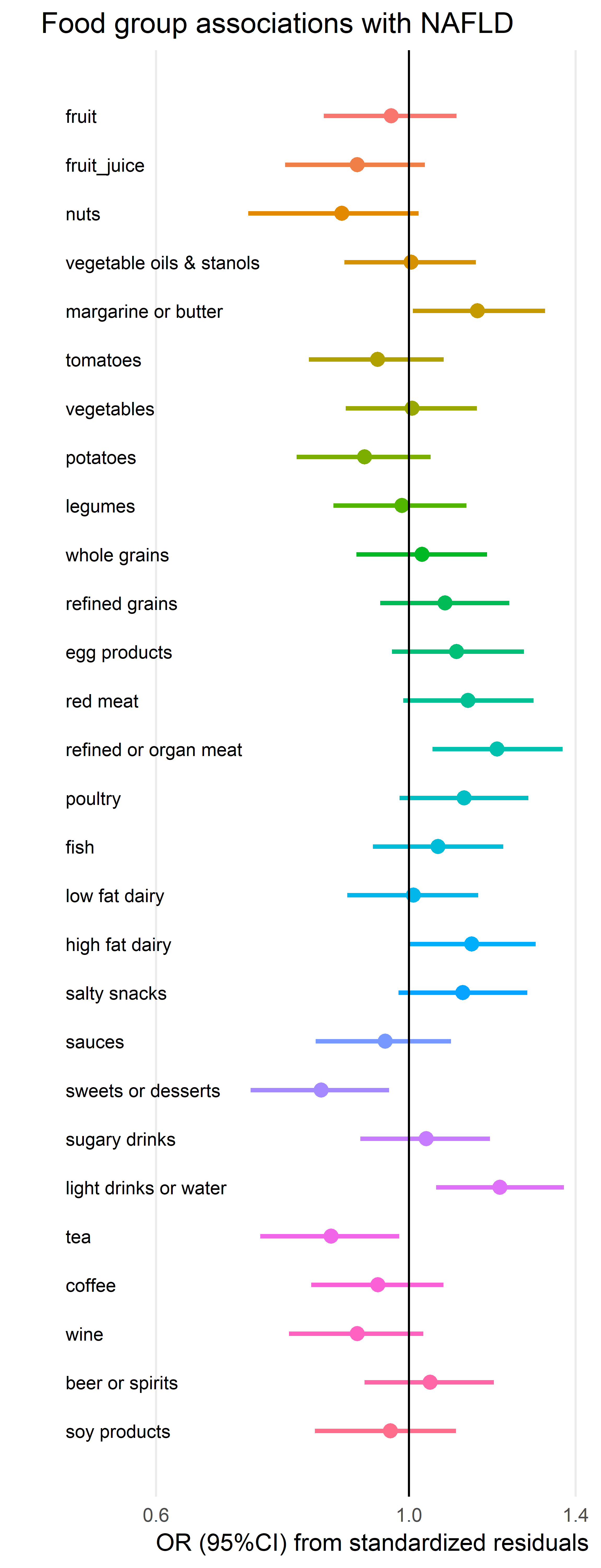


# Supplementary Figure 2: Food group univariable associations with NAFLD adjusted for energy intake.

This figure reflects the association between the different 28 food groups adjusted for energy intake using the residual method with NAFLD. The data is represented as odds ratio with 95% confidence interval. This figure represents cross-sectional data at baseline.

# Supplementary Table 3a: Complete logistic mixed model for risk of NAFLD with adherence to the MDS.

|  | **risk of NAFLD** |
| --- | --- |
|  | Full model |
|  | mean (95%CI) |
| follow-up time (years) | **0.73 [0.64, 0.83]** |
| age (years)* | 0.91 [0.81, 1.01] |
| sex (female) | **0.31 [0.09, 0.95]** |
| energy intake (kcals) | **1.00 [1.00, 1.00]** |
| Education level* |  |
| low | ref |
| intermediate | 0.41 [0.11, 1.36] |
| high | **0.17 [0.04, 0.72]** |
| physical activity* (metEqhs) | 1.00 [1.00, 1.00] |
| alcohol intake (units) | 1.08 [0.66, 1.80] |
| BMI (kg/m^2^) | **2.66 [2.11, 3.61]** |
| diabetes mellitus* | **29.4 [7.01, 161]** |
| hypertension* | **6.28 [1.49, 30.8]** |
| A-priori dietary patterns |  |
| MDS | 0.84 [0.66, 1.05] |

# Supplementary Table 3b: Complete logistic mixed model for risk of NAFLD with adherence to the DDG.

|  | **risk of NAFLD** |
| --- | --- |
|  | Full model |
|  | mean (95%CI) |
| follow-up time (years) | **0.74 [0.64, 0.83]** |
| age (years)* | 0.91 [0.81, 1.01] |
| sex (female) | **0.30 [0.09, 0.92]** |
| energy intake (kcals) | 1.00 [1.00, 1.00] |
| Education level* |  |
| low | ref |
| intermediate | 0.38 [0.10, 1.27] |
| high | **0.16 [0.03, 0.65]** |
| physical activity* (metEqhs) | 1.00 [0.99, 1.01] |
| alcohol intake (units) | 1.02 [0.61, 1.73] |
| BMI (kg/m^2^) | **2.69 [2.12, 3.70]** |
| diabetes mellitus* | **29.6 [6.78, 178]** |
| hypertension* | **6.47 [1.49, 34.5]** |
| A-priori dietary pattern |  |
| DDG | 0.89 [0.71, 1.12] |

# Supplementary Table 3c: Complete logistic mixed model for risk of NAFLD with adherence to the WHO.

|  | **risk of NAFLD** |
| --- | --- |
|  | Full model |
|  | mean (95%CI) |
| follow-up time (per year) | **0.73 [0.64, 0.82]** |
| age (per year) | 0.91 [0.81, 1.01] |
| sex (female) | 0.33 [0.10, 1.04] |
| energy intake (per kcal) | 1.00 [1.00, 1.00] |
| Education level* |  |
| low | ref |
| intermediate | 0.38 [0.11, 1.29] |
| high | **0.16 [0.03, 0.69]** |
| physical activity* (per metEqh) | 1.00 [0.99, 1.01] |
| alcohol intake (per unit) | 1.13 [0.68, 1.89] |
| BMI (per kg/m^2^) | **2.69 [2.13, 3.55]** |
| diabetes mellitus* | **29.7 [7.05, 161]** |
| hypertension* | **6.79 [1.56, 36.1]** |
| A-priori dietary pattern |  |
| WHO-score | **0.73 [0.53, 1.00]** |

These tables reflect the mixed logistic regression models for the three a-priori patterns (A: MDS, B: DDG, C: WHO). *Depicts baseline variables. Numbers in bold reflect a tail-probability of <0.05.

**Abbreviations:** BMI: body mass index, CI: credible interval, DDG: Dutch dietary guidelines, kcal: kilocalories, MDS: Mediterranean Diet Score, metEqh: metabolic equivalent hours, NAFLD: non-alcoholic fatty liver disease, WHO: world health organization.

# Supplementary Table 4a: Additional linear mixed regression between a-posteriori dietary patterns and BMI

|  | **BMI as outcome** |
| --- | --- |
|  | Full model |
|  | mean (95%CI) |
| follow-up time (years) | 0.01 [-0.01, 0.04] |
| age (years)* | -0.04 [-0.09, 0.01] |
| sex (female) | 0.13 [-0.37, 0.65] |
| energy intake (kcals) | 0.000 [0.000, 0.001] |
| Education level* |  |
| low | ref |
| intermediate | -0.17 [-0.71, 0.39] |
| high | **-0.85 [-1.54, -0.21]** |
| physical activity* (metEqhs) | **-0.008 [-0.013, -0.003]** |
| alcohol intake (units) | 0.04 [-0.10, 0.17] |
| diabetes mellitus* | **2.05 [1.38, 2.67]** |
| hypertension* | **1.43 [0.83, 2.03]** |
| A-posteriori dietary patterns |  |
| Vegetable & Fish Pattern | 0.002 [-0.22, 0.24] |
| Red Meat & Alcohol Pattern | 0.15 [-0.07, 0.36] |
| Traditional Pattern | -0.09 [-0.31, 0.14] |
| Salty Snacks & Sauces Pattern | 0.07 [-0.16, 0.29] |
| High Fat Dairy & Refined Grains Pattern | -0.09 [-0.22, 0.03] |
| Interaction terms |  |
| FU-time × Vegetable & Fish Pattern | -0.004 [-0.06, 0.05] |
| FU-time × Red Meat & Alcohol Pattern | -0.02 [-0.07, 0.05] |
| FU-time × Traditional Pattern | -0.02 [-0.06, 0.01] |
| FU-time × Salty Snacks & Sauces Pattern | 0.01 [-0.04, 0.05] |
| FU-time × High Fat Dairy & Refined Grains Pattern | **-0.04 [-0.07, -0.01]** |

# Supplementary Table 4b: Additional linear mixed regression between MDS and BMI

|  | **BMI as outcome** |
| --- | --- |
|  | Full model |
|  | mean (95%CI) |
| follow-up time (years) | 0.001 [-0.08, 0.08] |
| age (years)* | -0.05 [-0.10, 0.001] |
| sex (female) | 0.06 [-0.44, 0.57] |
| energy intake (kcals) | 0.000 [0.000, 0.000] |
| Education level* |  |
| low | ref |
| intermediate | -0.18 [-0.73, 0.39] |
| high | **-0.87 [-1.52, -0.21]** |
| physical activity* (metEqhs) | **-0.008 [-0.013, -0.003]** |
| alcohol intake (units) | 0.04 [-0.09, 0.18] |
| diabetes mellitus* | **2.08 [1.43, 2.77]** |
| hypertension* | **1.46 [0.82, 2.10]** |
| A-priori dietary patterns |  |
| MDS | -0.005 [-0.08, 0.06] |
| Interaction terms |  |
| FU-time × MDS | 0.003 [-0.01, 0.018] |

# Supplementary Table 4c: Additional linear mixed regression between DDG and BMI

|  | **BMI as outcome** |
| --- | --- |
|  | Full model |
|  | mean (95%CI) |
| follow-up time (years) | -0.104 [-0.21, 0.002] |
| age (years)* | -0.05 [-0.10, 0.003] |
| sex (female) | 0.06 [-0.46, 0.58] |
| energy intake (kcals) | 0.000 [0.000, 0.000] |
| Education level* |  |
| low | ref |
| intermediate | -0.18 [-0.75, 0.40] |
| high | **-0.89 [-1.55, -0.24]** |
| physical activity* (metEqhs) | **-0.008 [-0.01, -0.003]** |
| alcohol intake (units) | 0.05 [-0.09, 0.18] |
| diabetes mellitus* | **2.06 [1.43, 2.71]** |
| hypertension* | **1.47 [0.83, 2.09]** |
| A-priori dietary patterns |  |
| DDG | -0.02 [-0.09, 0.04] |
| Interaction terms |  |
| FU-time × DDG | **0.016 [0.002, 0.031]** |

# Supplementary Table 4d: Additional linear mixed regression between WHO and BMI

|  | **risk of NAFLD** |
| --- | --- |
|  | Full model |
|  | mean (95%CI) |
| follow-up time (years) | -0.09 [-0.17, 0.003] |
| age (years)* | -0.05 [-0.10, 0.003] |
| sex (female) | 0.07 [-0.44, 0.58] |
| energy intake (kcals) | 0.000 [0.000, 0.000] |
| Education level* |  |
| low | ref |
| intermediate | -0.18 [-0.75, 0.36] |
| high | **-0.87 [-1.52, -0.21]** |
| physical activity* (metEqhs) | **-0.01 [-0.01, -0.003]** |
| alcohol intake (units) | 0.05 [-0.09, 0.18] |
| diabetes mellitus* | **2.07 [1.40, 2.72]** |
| hypertension* | **1.47 [0.87, 2.08]** |
| A-priori dietary patterns |  |
| WHO | -0.089 [-0.17, 0.002] |
| Interaction terms |  |
| FU-time × WHO | **0.03 [0.01, 0.05]** |

These tables show the results (mean estimate and 95% CIs) of multivariable linear mixed models f a posteriori dietary patterns (A) and a priori dietary patterns (B-D). *Depicts baseline variables. Numbers in bold reflect a tail-probability of <0.05.

**Abbreviations:** BMI: body mass index, CI: credible interval, DDG: Dutch Dietary Guidelines, MDS: Mediterranean Diet Score, NAFLD: non-alcoholic fatty liver disease, kcal: kilocalories, metEqh: metabolic equivalent hours, WHO: World Health Organization.


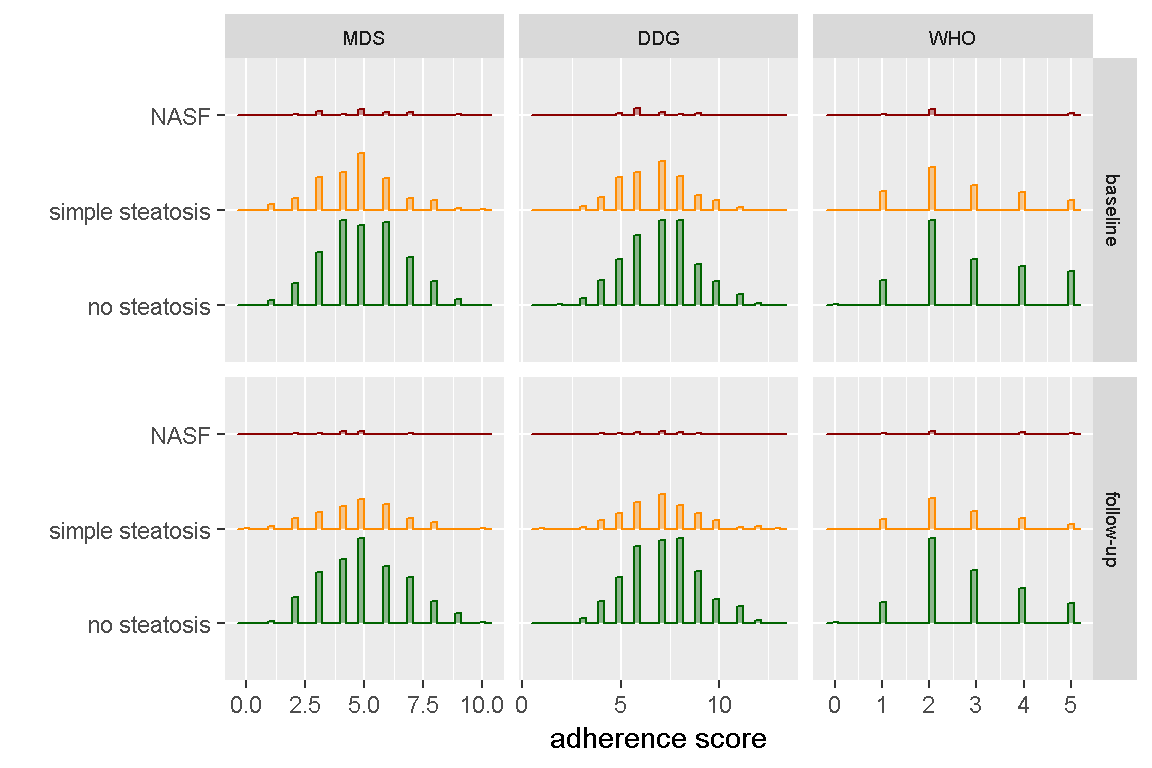


# Supplementary Figure 3a: Visualization of adherence scores to the a-priori dietary patterns across participants without NAFLD, with simple steatosis, and NASF.


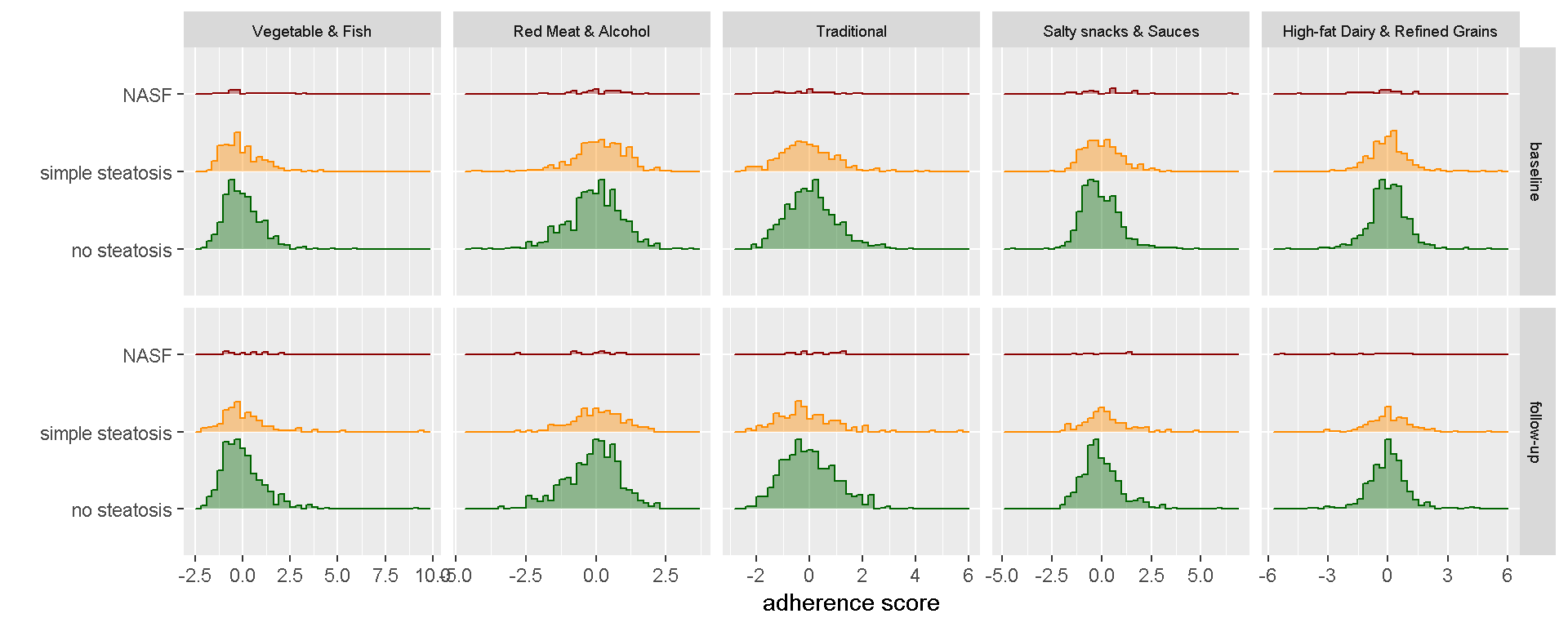


# Supplementary Figure 3b: Visualization of adherence scores to the a-posteriori dietary patterns across participants without NAFLD, with simple steatosis, and NASF.

These figures represent the absolute number of individuals per adherence score (on the x-axis) against NAFLD severity on the y-axis (i.e. no steatosis, simple steatosis: steatosis without elevated liver stiffness, and non-alcoholic steatofibrosis: steatosis with elevated liver stiffness) illustrated per dietary pattern (A: a priori dietary patterns, B: a posteriori dietary patterns).

**Abbrevations:** DDG: Dutch dietary guideline, MDS: Mediterranean diet score, NASF: non-alcohol steatofibrosis, WHO: world health organization
