# Supplementary material for: Adherence to a plant-based, high-fibre dietary pattern is related to regression of non-alcoholic fatty liver disease in an elderly population
Source: Eur J Epidemiol. 2020 Apr 22;35(11):1069–85. doi: 10.1007/s10654-020-00627-2 (PMC7695656; doi:10.1007/s10654-020-00627-2)
Supplement: Supplementary file 2 — Supplementary material 2 (PDF 273 kb) [file 10654_2020_627_MOESM2_ESM.pdf]

Adherence to a plant-based, high-fibre dietary pattern is related to regression of non-alcoholic fatty liver disease in an elderly population

Supplementary Material: Missing Data & Imputed Values

Louise J.M. Alferink[1], Nicole S. Erler[2], Robert J. de Knegt[1], Harry L.A. Janssen[3], Herold J. Metselaar[1], Sarwa Darwish Murad[1], Jessica C. Kiefte-de Jong[4,5]

[1]Department of Gastroenterology and Hepatology, Erasmus Medical Center, Rotterdam, The Netherlands

[2]Department of Biostatistics, Erasmus Medical Center, Rotterdam, The Netherlands

[3]Toronto Centre of Liver Disease, Toronto General Hospital, University Health Network, Toronto, Canada

[4]Department of Epidemiology, Erasmus Medical Center, Rotterdam, The Netherlands

[5]Department of Public Health and Primary Care, Leiden University Medical Center, The Hague, The Netherlands

Missing Values

Figure 1 visualizes the pattern of missing values at baseline. Out of the 963 cases used in the analysis, 868 had complete information at baseline. There were 68 cases for whom the physical activity was unknown, 26 cases with unobserved education level and 1 case with missing BMI at baseline.

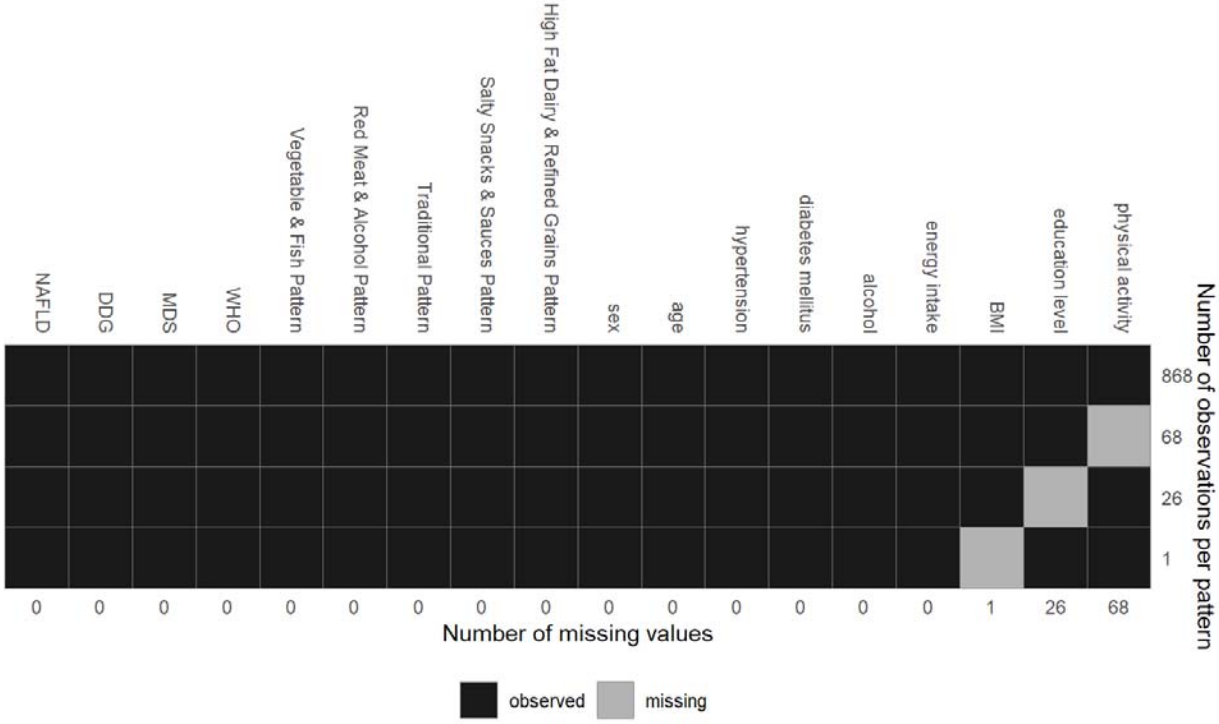

Figure 1: Missing data pattern of all variables that are measured at baseline.

The distribution of these missing values across the outcome groups is shown in Table 1.

Table 1: Number of missing observations by outcome group, and proportion relative to the number of observations in the respective outcome group.

	**no NAFLD (N=620)**	**NAFLD (N=343)**
physical activity	39 (6.3%)	29 (8.5%)
education level	15 (2.4%)	11 (3.2%)
BMI	1 (0.2%)	0 (0.0%)

At follow-up, 323 cases had dropped out (non of them was registered as deceased), leaving 640 cases with data available at this time point. There were no missing values in any of the variables measured at follow-up.

Imputed Values

Missing values were imputed in the Bayesian framework. The missing values in BMI, physical activity and education level were imputed, conditional on other covariates, from a normal distribution, gamma distribution with a log-link, and a categorical distribution, respectively. A cumulative logit link was used in the model for educational level to take into account the ordinal structure of this variable.

Figures 2 - 5 show the empirical distribution of the original data and the distribution of the ten imputed values (excluding the observed values) that were used in subsequent analyses. For physical activity, each thin line shows the distribution of the imputed values for all cases with missing physical activity from one imputed dataset (i.e., one line for each of the ten imputations). For BMI, where there was only one value missing, the thin line shows the distribution over all then imputed values, and each of the imputed values is represented by a dot. For education level, the first (darker) bar represents the observed data, all other bars show the distribution of imputed values for each of the ten sets of imputed values.

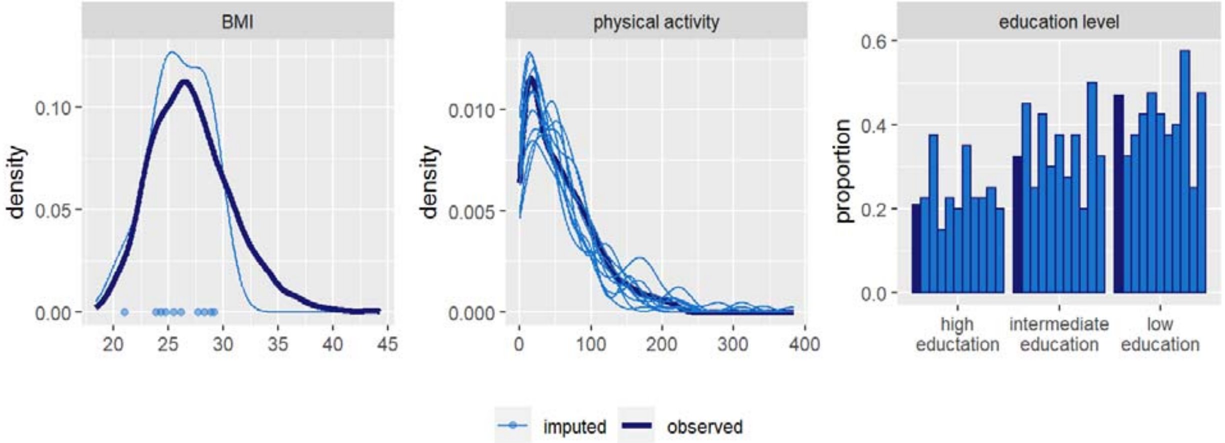

Figure 2: Distribution of observed and imputed values from the model in which diet was measured by the DDG pattern.

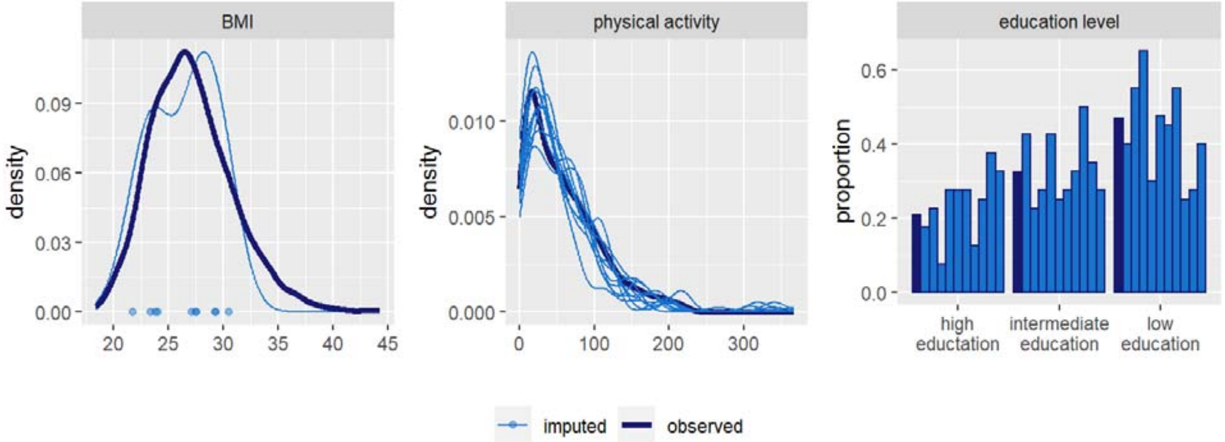

Figure 3: Distribution of observed and imputed values from the model in which diet was measured by the MDS pattern.

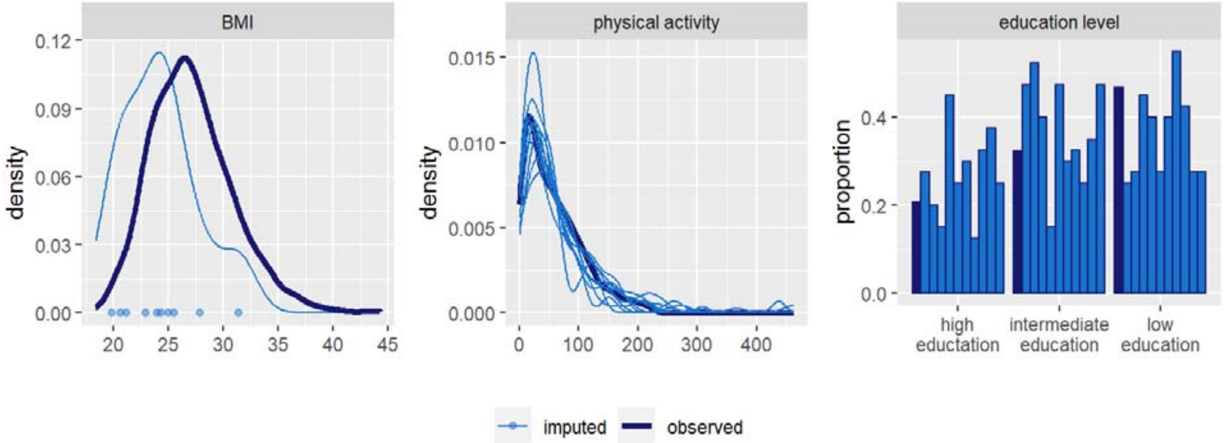

Figure 4: Distribution of observed and imputed values from the model in which diet was measured by the WHO pattern.

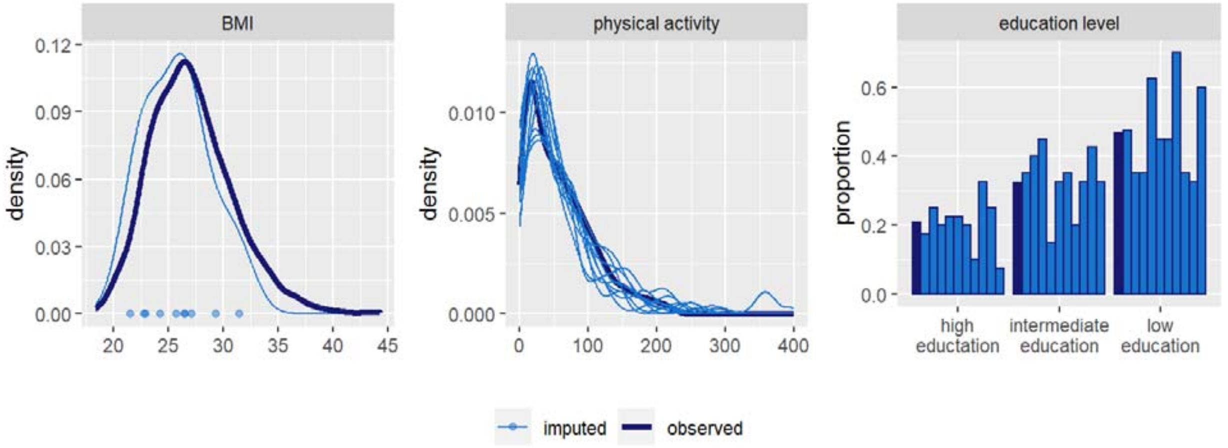

Figure 5: Distribution of observed and imputed values from the model in which diet was measured by the *a-posteriori* dietary patterns.

Overall, the distribution of the imputed values is similar to the distribution of the observed data. It is important to note that Figures 2 - 5 show the marginal distributions. The distributional assumptions made in the models are, however, conditional on the values of other covariates, which can not be visualized as easily.

The difference that is seen in the distribution for the observed and imputed values for BMI (especially in the model using the WHO pattern, Figure 4), can partially be explained by the small number of data points available for the kernel density estimation used in this visualization, and could potentially be explained by the values of the covariates used in the model for BMI. Since there is only one value of BMI missing, the impact of the imputed values for BMI on the results is in any case negligible.
